# Comprehensive Analysis of the Expression of Key Genes Related to Hippo Signaling and Their Prognosis Impact in Ovarian Cancer

**DOI:** 10.3390/diagnostics11020344

**Published:** 2021-02-19

**Authors:** Paul Kubelac, Cornelia Braicu, Lajos Raduly, Paul Chiroi, Andreea Nutu, Roxana Cojocneanu, Liviuta Budisan, Ioana Berindan-Neagoe, Patriciu Achimas-Cadariu

**Affiliations:** 1 Department of Oncology, Iuliu Hatieganu University of Medicine and Pharmacy, 400012 Cluj Napoca, Romania; paulkubelac@yahoo.com; 2 Department of Oncology, The Oncology Institute “Prof. Dr. Ion Chiricuta”, 400015 Cluj Napoca, Romania; 3 Research Center for Functional Genomics, Biomedicine and Translational Medicine, Iuliu Hatieganu University of Medicine and Pharmacy, 400337 Cluj-Napoca, Romania; raduly.lajos78@gmail.com (L.R.); chiroipaul@gmail.com (P.C.); andreeanutu.an@gmail.com (A.N.); cojocneanur@gmail.com (R.C.); lbudisan@yahoo.com (L.B.); 4 Department of Surgery, Institute of Oncology “Prof. Dr. Ion Chiricuta", 400015 Cluj-Napoca, Romania; pachimas@umfcluj.ro; 5 Department of Surgery and Gynecological Oncology, Iuliu Hatieganu University of Medicine and Pharmacy, 400337 Cluj-Napoca, Romania

**Keywords:** ovarian cancer, Hippo signaling, gene expression, miRNA, prognostic value

## Abstract

The Hippo signaling pathway, one of the most conserved in humans, controlling dimensions of organs and tumor growth, is frequently deregulated in several human malignancies, including ovarian cancer (OC). The alteration of Hippo signaling has been reported to contribute to ovarian carcinogenesis and progression. However, the prognostic roles of individual Hippo genes in OC patients remain elusive. Herein we investigated the expression level and prognostic value of key Hippo genes in OC using online databases, followed by a qRT-PCR validation step in an additional patient cohort. Using the GEPIA database, we observed an increased level for TP53 and reduced expression level for LATS1, LATS2, MST1, TAZ, and TEF in tumor tissue versus normal adjacent tissue. Moreover, LATS1, LATS2, TP53, TAZ, and TEF expression levels have prognostic significance correlated with progression-free survival. The qRT-PCR validation step was conducted in an OC patient cohort comprising 29 tumor tissues and 20 normal adjacent tissues, endorsing the expression level for LATS1, LATS2, and TP53, as well as for two of the miRNAs targeting the TP53 gene, revealing miR-25-3p upregulation and miR-181c-5p downregulation. These results display that there are critical prognostic value dysregulations of the Hippo genes in OC. Our data demonstrate the major role the conserved Hippo pathway presents in tumor control, underlying potential therapeutic strategies and controlling several steps modulated by miRNAs and their target genes that could limit ovarian cancer progression.

## 1. Introduction

Ovarian cancer is usually diagnosed in advanced stages due to its nonspecific symptomatology and is the deadliest gynecological malignancy. Over 70% of cases are diagnosed with FIGO stage III or IV disease, where the five-year survival rate is 25% [1,2,3,4,5]. Numerically speaking, in 2018, the worldwide incidence of ovarian cancer (OC) increased to 295,414 cases, and the mortality followed with not less than 184,799 deaths [6,7]. Therefore, OC is the seventh most frequent type of cancer and the eighth most common cause of cancer death in women, with a case-to-fatality ratio nearly three times higher than breast cancer [2,3,8].

Clinical genomics promises unprecedented precision in the comprehension of the genetic basis of cancers, including the case of OC, being proved the important role of the coding and non-coding genes [8,9,10]. Among the non-coding genes, miRNAs as short transcripts (19–25 bp in length) modulate transcriptional and implicitly translational programs and therefore orchestrate key cellular processes [11,12]. They were identified as aberrantly expressed in a wide range of pathologies, having prognostic value for many cancers [12,13,14]. miRNAs also regulate the main pathways that regulate cancer progression [13,15,16]. Among them, nuclear receptor, RTK, Hippo, Notch, and Wnt/B-catenin pathways have shown a notable involvement [15,17].

The Hippo signaling pathway is an evolutionarily conserved protein kinase cascade that regulates organ size in the course of development from flies to humans [16]. This highly conserved signaling pathway [18] regulates multiple growth and development processes, including cellular proliferation and apoptosis, stem cell self-renewal and differentiation, tissue homeostasis, and organ size [19,20]. Increasingly more evidence confirmed this pathway’s involvement in cancer progression and metastasis, gaining considerable interest as a major player in cancer biology [20,21]. There is enough proof to indicate that dysregulation in the expression of Hippo pathway components is strongly correlated with many cancer hallmarks, leading to a poor prognosis in a broad range of human malignancies, including OC [20]. Whereas Hippo is ruled by several specific genes and their level of expression, those are modulated by miRNAs, as stated above.

Hippo signaling acts as an ancient mechanism preceding the emergence of multicellularity. This evolutionarily conserved kinase cascade culminates in the phosphorylation of YAP and TAZ, the mammalian counterpart of Yki [22]. Conserved Hippo pathway targets in both *Drosophila* and mammals signify that Yki/YAP/TAZ can persuade other oncogenic transcriptional factors to further boost their oncogenic activity [23].

The core of the Hippo pathway is represented by a kinase cascade consisting of Ste20-like protein kinase 1 (STK3/MST2 and STK4/MST1), the tumor suppressors LATS1 and LATS2, and adaptor proteins Salvador homolog 1 (SAV1) along with the MOB kinase activators (MOB1A/MOB1B) [24]. The yes-associated protein (YAP) is one of the downstream regulatory proteins in the Hippo, directly related to the transcriptional coactivator with PDZ-binding motif (TAZ) [25]. YAP/TAZ is translocated in the nucleus and interacts majorly with TEA domain family member (TEAD) transcription factors (TEAD1-4), activating the transcription mechanism for key genes involved in the regulation of the cell fate [25] and promoting epithelial to mesenchymal transition (EMT), stem cells features, or invasion and metastasis [26]. However, the prognostic roles of individual key Hippo signaling related genes, especially at the mRNA level in OC patients, remain elusive. In the current study, we accessed the expression level using GEPIA and UALCAN interactive web servers analyzing OMICS data [27,28].

We checked for the prognostic role of these genes in OC patients by the Kaplan–Meier plotter (KM plotter). KM plotter generated data from the Gene Expression Omnibus (GEO: www.ncbi.nlm.nih.gov/geo/, accessed on 4 January 2021) database [29]. KM plotters analyze individual genes with clinical results for relapse-free survival and total survival of the patients. Progression-free survival is a desirable outcome because it is not influenced by later-line therapies and can be measured earlier than overall survival (OS) [30]. So far, several genes have been identified and/or validated by the KM plotter.

In this study, we used the KM plotter database and accessed the prognostic roles of Hippo signaling genes mRNA expression in OC patients. Additionally, we aimed to screen potential key Hippo signaling related genes and their direct related miRNA, targeting these genes involved in the pathogenesis and prognostic markers through integrated bioinformatics analysis on OC, followed by a validation step for key miRNAs (miR-25 and miR-181c) and key Hippo tumor suppressor genes (LATS1, LATS2, and TP53).

## 2. Materials and Methods

### 2.1. Gene Expression Profiling Interactive Analysis of the Key Hippo Genes

We performed the analysis of gene expression profiling on OC samples using the GEPIA database (http://gepia.cancer.pku.cn/, accessed on 4 January 2021) and the UALCAN database (http://ualcan.path.uab.edu/, accessed on 4 January 2021). The functions of the UALCAN database for OC are divided into expression analysis and custom data analysis according to race, age, tumor grade, and TP53 mutation status. This database includes gene expression data and survival information from a total of 1435 OC patients. The KM plotter analyzes individual genes against clinical outcomes such as relapse-free survival (PFS) and overall survival (OS). PFS is a beneficial outcome considering that it is not affected by later-line therapies and can be measured earlier than OS [31].

### 2.2. Survival Outcome Analysis of OC Patients

We used an online database (http://kmplot.com/analysis/, accessed on 4 January 2021) to determine the significance of gene expression in PFS. Six Hippo signaling genes: LATS1 and LATS2 (both tumor suppressor genes), MST1 (mammalian Ste20-like kinases 1), tumor suppressor gene TP53, transcriptional coactivator with PDZ-binding motif, TAZ, and TEF from TEAD/TEF family of transcription factors were entered into the database (https://kmplot.com/analysis/index.php?p=service, accessed on 4 January 2021) to attain Kaplan–Meier survival plots in which the number-at-risk is specified under the main plot by selecting the JetSet best probe set [32]. We calculated the hazard ratio (HR) and 95% confidence intervals and log rank; we considered a *p*-value of <0.05 to be statistically significant.

### 2.3. miRNA and Gene Validation in OC Samples

A total of 29 histologically confirmed OC patients admitted at The Oncology Institute Prof. Dr. Ion Chiricuta Cluj-Napoca, Romania, during 2018–2020 were enrolled in this study after the approval of the ethical committee. All patients signed the informed consent. The age of patients ranged between 22–75 years. All patients were staged according to the American Joint Committee on Cancer (AJCC) guidelines. Immediately following surgical excision, all tissue samples were snap-frozen in liquid nitrogen for RNA isolation and stored at −80 °C. Patients’ clinical data are presented in Table 1.

We extracted total RNA from tumors (*n* = 29) and adjacent normal tissues (*n* = 20) using TriReagent (Ambion, Austin, TX, USA), according to the manufacturer’s instructions. We used 1000 ng of total RNA for reverse transcription into cDNA using a High-Capacity cDNA Reverse Transcription Kit (Applied Biosystems, Foster City, CA, USA). The final step of gene expression protocol was amplification using SYBR Select Master Mix on ViiA^TM^7 System, using specific primers for target genes (LATS1: GGCACAAACACCATTAGAAACA/AGAAGCTTCAGGACTGAGTTTAGC; LATS2: AGCAAGAAATGGCCAAAGC/GGTAGAGGATCTTCCGCATCT; TP53: CCCTTTTTGGACTTCAGGT/AGGCCTTGGAACTCAAGGAT) and housekeeping genes (B2M: CACCCCCACTGAAAAAGATGAG/CCTCCATGATGCTGCTTACATG). The miRNA gene expression protocol was started from 50 ng of total RNA for reverse transcription reaction using a TaqMan MicroRNA Reverse Transcription Kit (Applied Biosystems), the amplification using TaqMan Fast Advanced Master Mix (Applied Biosystems) and TaqMan assays (U48: 001006; U6: 001973; miR-25: 000403; miR-181c: 000482) on the same instrument. The relative quantification of the gene and miRNA expression levels was conducted using the 2^−ΔΔCT^ method [33].

## 3. Results

### 3.1. Transcriptional Levels of Main Representative Members of Hippo Signaling in Patients with OC

**Figure 1:**
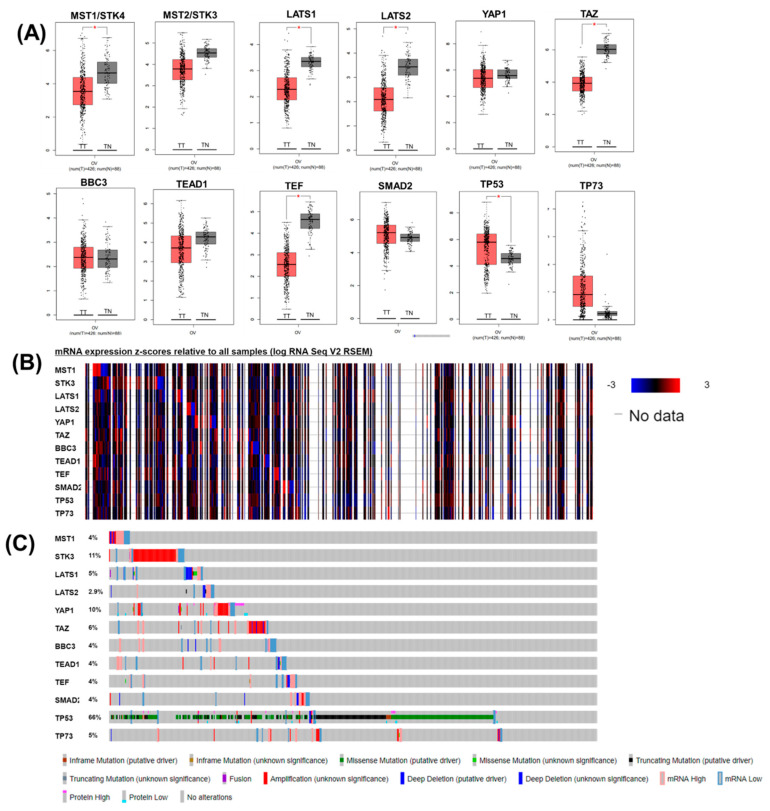
Hippo pattern in OC. (**A**) The expression level of the main genes, Hippo genes, upstream effectors (MST1/ STK4, MST2/STK3, LATS1/2), YAP/TAZ, and downstream genes (BBC3, TEAD1, SMAD2, TP53, and TP73) involved in Hippo signaling in OC (box plot representation using GEPIA, TT: tumor tissues, TN: normal tissues); (**B**) Heatmap of mRNA expression results by querying the interest genes with the default setting (*z*-score threshold of 2) generated using cBioPortal for ovarian serous cystadenocarcinoma (TCGA, PanCancer Atlas, 585 patients); (**C**) Gene-related changes in critical components of the Hippo pathways in OC. Mutational pattern for selected genes on the same patient cohort. (* *p* value ≤ 0.05).

Several genes related to Hippo signaling were identified in mammalian cells; among them, we selected 12 for analysis of the expression level. We compared the transcriptional levels of the main representative members of Hippo signaling within OC using the GEPIA database. We observed a statistically significant increased level for TP53 and a reduced expression level for LATS1, LATS2, MST1, TAZ, and TEF (Figure 1A). The expression levels heatmap generated using cBioPortal is presented in Figure 1B. We observed a higher mutation rate for TP53 (66%), STK3 (11%), YAP1 (10%), and a less frequent mutation rate for LATS1, LATS2, MST1, TAZ, and TEF (Figure 1C). LATS1 and TAZ expression levels decreased with stage, and we observed no significant differences in subgroup analysis for tumor grade and TP53 mutational status (Figure S1).

### 3.2. Association between Gene Expression and Progression-Free Survival (PFS) in OC

**Figure 2:**
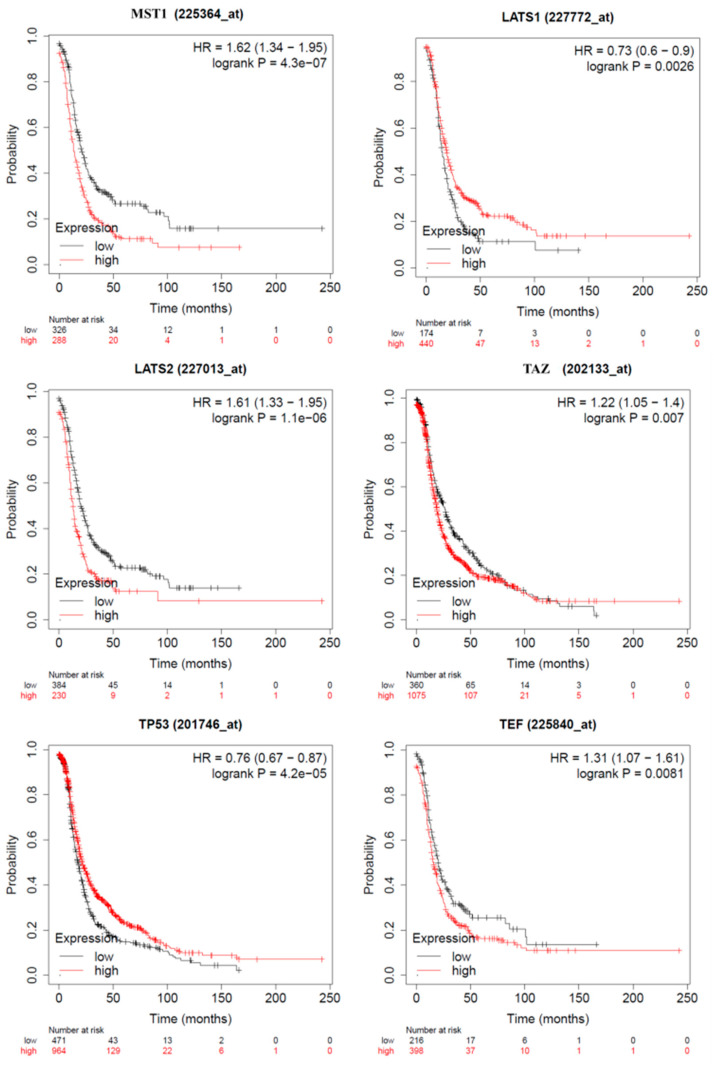
Progression-free survival (PFS) of key Hippo signaling genes for OC patients, according to high or low expression level. *n* = 1435 OC patients according to the expression of the Kaplan–Meier plotter (https://kmplot.com/analysis/index.php?p=service, accessed on 4 January 2021) based on TCGA patients, a log-rank test was used (MST1 high/low, *p* = 0.1; LATS1 high/ low *p* = 0.0026; LATS2 low/high *p* = 1.1 × 10^−6^; TAZ low/high, *p* = 0.007; TEF high/low, *p* = 0.0081; TP53 high/low, *p* = 4.2 × 10^−5^).

To determine whether Hippo signaling gene expression is associated with prognosis in OC, we analyzed PFS between the high- and low-gene expression groups from the KM plotter online database. The results indicated that except for MST1, expression levels for LATS1, LATS2, TP53, TAZ, and TEF had a significant impact on PFS (*p* < 0.05, Figure 2). High expression of MST1, LATS1, and TP53 was associated with a better prognostic, whereas low expression of LATS2, TAZ, and TEF was associated with a better prognostic in OC.

**Figure 3:**
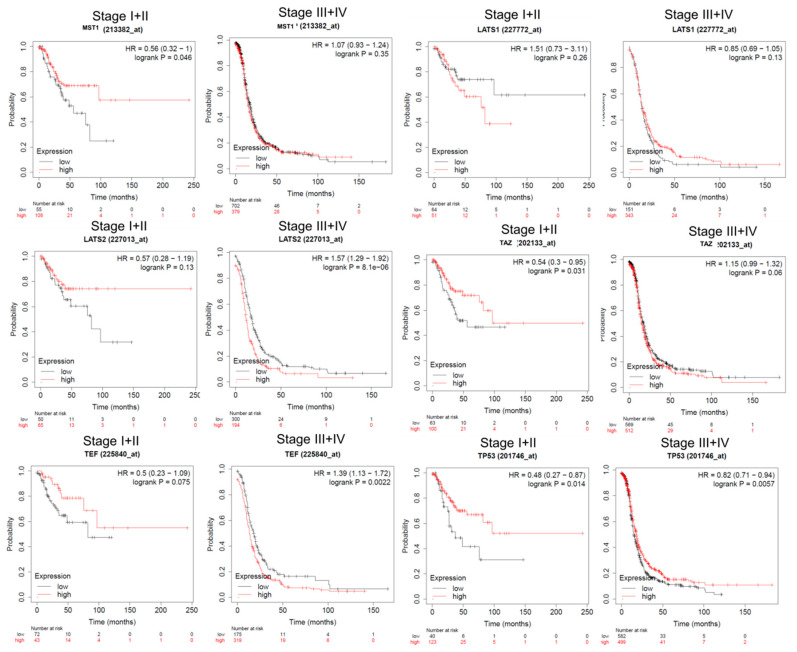
Progression-free survival (PFS) subgroup analysis of key Hippo signaling genes for OC patients, according to high or low expression level based on tumor stage. PFS of key Hippo signaling genes (MST1, LATS1, LATS2, TAZ, MST1, and TEF) for OC patients, grouped on stage I + II and III + IV, respectively. Survival curves of OC patients according to the expression of the Kaplan–Meier plotter (https://kmplot.com/analysis/index.php?p=service, accessed on 4 January 2021) based on TCGA patients, a log-rank test was used (LATS1 high/low, I + II *p* = 0.26 and III + IV *p* = 0.13; TP53 high/low I + II *p* = 0.014 and III + IV *p* = 0.0057; LATS2 low/high, I + II *p* = 0.13 and III + IV *p* = 8.1 × 10^−6^; TAZ low/high I + II *p* = 0.031 and III + IV *p* = 0.06; MST1 low/high, I + II *p* = 0.046 and III + IV *p* = 0.35; TEF high/low, I + II *p* = 0.075 and III + IV *p* = 0.0022).

Subgroup analysis on tumor stage for LATS1 was not statistically significant. Low expression of LATS2 and TEF in advanced stages (III + IV) was associated with a significantly better prognostic; however, this was not significant for early stage OC (I + II). High expression of TAZ and MST1 was associated with a significantly better prognostic in early stage OC (I + II) but not in advanced stage OC (III + IV). High expression of TP53 was associated with a significantly better prognostic in both early (stage I + II) and advanced (stage III + IV) OC (Figure 3 and Table 2).

**Figure 4:**
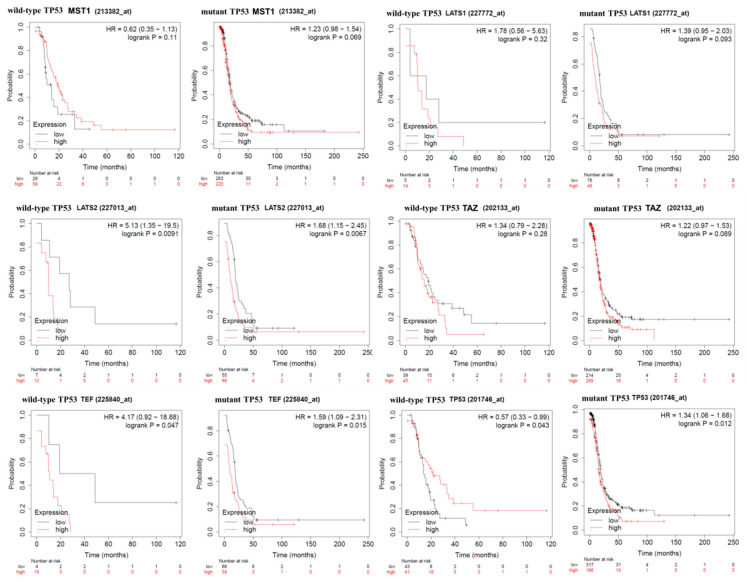
Progression-free survival (PFS) subgroup analysis of key Hippo signaling genes for OC patients, according to high or low expression level based on TP53 status. PFS analysis of key Hippo signaling genes (MST1, LATS1, LATS2, TAZ, MST1, and TEF) for OC patients separated on TP53 status. PFS survival curve of OC patients according to the expression of Kaplan–Meier plotter (https://kmplot.com/analysis/index.php?p=service, accessed on 4 January 2021) based on TCGA patients, a log-rank test was used for TP53 mutated and TP53 wild-type (LATS1 high/low, wild-type TP53 *p* = 0.32 and mutant TP53 *p* = 0.093; TP53 high/low, wild-type TP53 *p* = 0.03 and mutant TP53 *p* = 0.012; LATS2 high/low, wild-type TP53 *p* = 0.0091 and mutant TP53 *p* = 0.0067; TAZ high/low, wild-type TP53 *p* = 0.26 and mutant TP53 *p* = 0.89; MST1 high/low, wild-type TP53 *p* = 0.11 and mutant TP53 *p* = 0.69; TEF high/low, wild-type TP53 *p* = 0.047 and mutant TP53 *p* = 0.015).

High expression of TP53 wild-type was associated with a favorable prognostic, whereas high expression of TP53 mutated was associated with an unfavorable prognostic. Subgroup analysis on TP53 mutational status revealed that MST1, LATS1, and TAZ were not associated with PFS. Low expression of LATS2 and TEF were associated with a significantly longer PFS rate in both groups (TP53 mutant and TP53 wild-type, Figure 4 and Table 3).

### 3.3. miRNA-mRNA Interaction Network Analysis via miRNET

**Figure 5:**
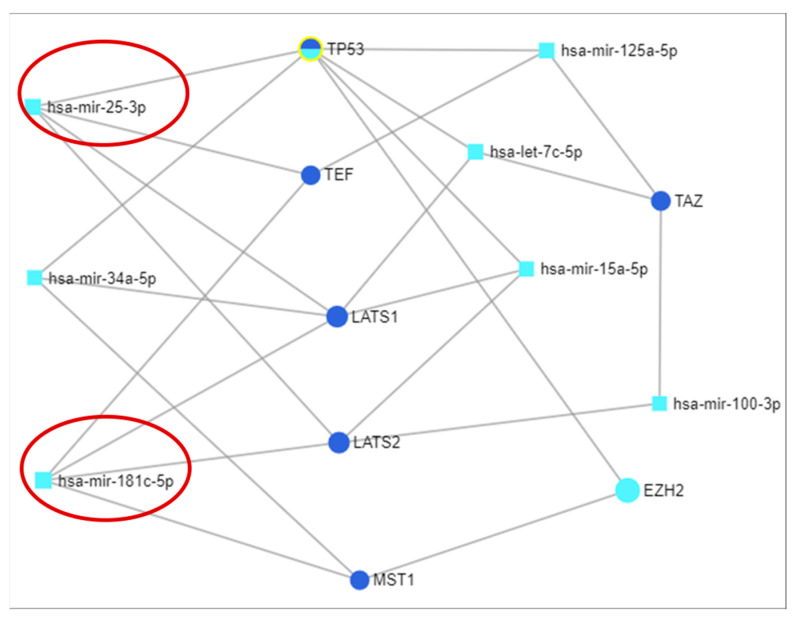
mRNA-miRNA interconnected network generates using miRNET. Red circles are highlighted miRNA selected for qRT-PCR validation.

The miRNA-mRNA interaction network revealed a correlation among genes involved in Hippo signaling, uncovering the high complexity of biological mechanisms, with each gene being targeted by a number of important miRNAs. Figure 5 presents the extracted miRNA-mRNA network, emphasizing those miRNAs that target at least three genes from the selected Hippo genes. The core of the network is TP53, interconnected with one of the most powerful tumor suppressor miRNAs, namely miR-34a-5p (known in literature to be related with OC prognostic), along with miR-25-3p, let-7c-5p, miR-15a-5p, and miR-125a-5p.

### 3.4. Validation of Key miRNA Related to Hippo Signaling by qRT-PCR

**Figure 6:**
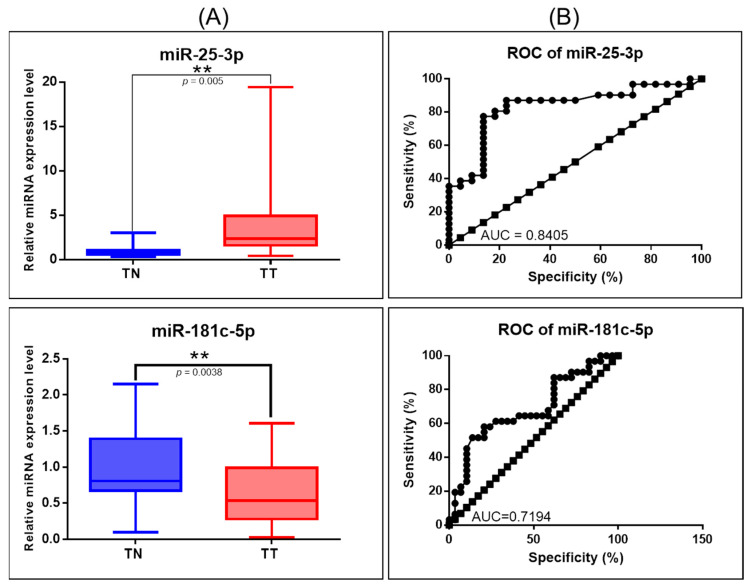
miRNA targeting key Hippo signaling genes in OC. (**A**) Scatter plots demonstrating the upregulation of miR-25-3p and downregulation of miR-181c-5p in tumor tissues versus normal adjacent tissues. We used for normalization of the miRNA expression data, U6 and RNU48 as the internal controls (** *p* ≤ 0.01); (**B**) ROC curve for miR-25-3p and miR-181c-5p.

For the validation step, we selected the TP53 targets: miR-25-3p and miR-181c-5p. Validation of miRNAs was conducted using 29 tissue samples collected from OC, as well as 20 samples of distant normal tissues. For normalization of the miRNA data, U6 and RNU48 were used as internal controls, based on the ΔΔCt method. miR-25-3p levels were overexpressed, whereas miR-181c-5p was downregulated (Figure 6A). Additionally, a ROC (receiver-operating characteristic) curve was generated to assess the sensitivity and specificity of these genes, the highest AUC (area under the curve) value being for miR-25b-3p (0.8405) (Figure 6B).

### 3.5. Validation of Key Genes Related to Hippo Signaling by qRT-PCR

**Figure 7:**
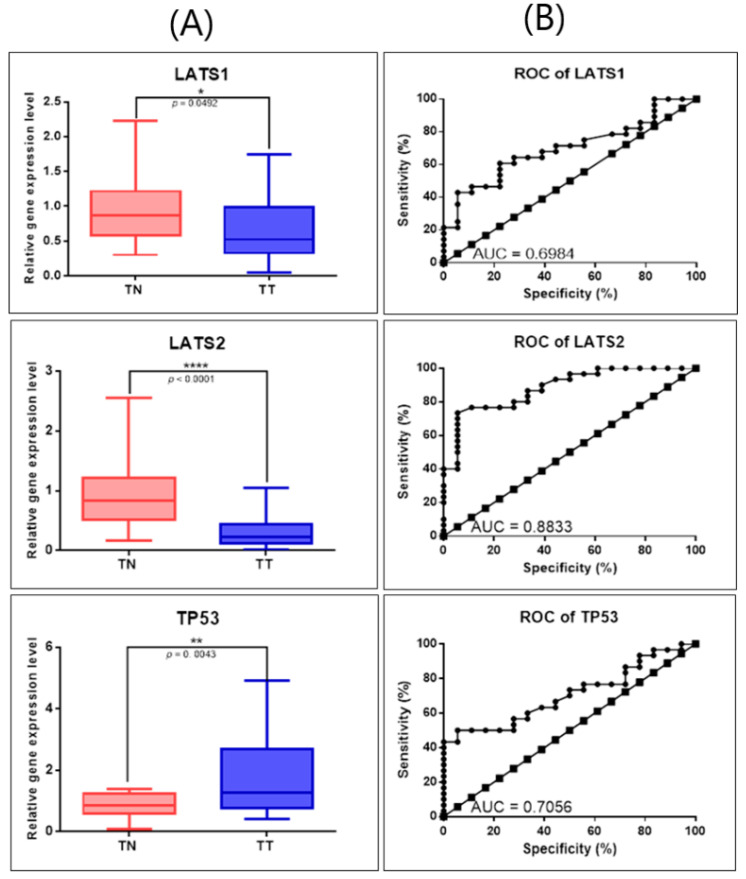
Gene expression alterations in OC evaluated by qRT-PCR. (**A**) Scatter plots demonstrating the downregulation of LATS1 and LATS2 and upregulation of TP53 in tumor tissues (TT, *n* = 30) versus normal tissues (TN, *n* = 22). For normalization of the gene expression data, we used B2M as internal control (* *p* ≤ 0.05, ** *p* ≤ 0.01, **** *p* ≤ 0.0001); (**B**) ROC curves for each selected gene’s specificity and sensitivity (ROC: receiver-operating characteristic, AUC: area under ROC curve).

Validation of selected genes was conducted on the same patient cohort used for the evaluation of miRNAs. In order to further validate the gene expression alteration revealed using the GEPIA online tool, we performed qRT-PCR for LATS1, LATS2, and TP53, with the B2M gene as the endogenous control for normalization of the qRT-PCR data. Gene expression analysis showed that TP53 is overexpressed, whereas LATS1 and LATS2 levels were significantly underexpressed in tumor tissues versus normal adjacent tissues (Figure 7A). For each evaluated gene, we generated a ROC curve to assess the sensitivity and specificity of these genes, the highest AUC value being for LATS1 (0.6984), LATS2 (0.8833), and TP53 (0.7056) (Figure 7B). These qRT-PCR results further validate our earlier gene and miRNA expression profiles from the GEPIA database.

## 4. Discussion

Ovarian cancer still has the highest mortality among gynecological malignancies. Although novel strategies for OC treatment continue to emerge, the effectiveness of novel treatments remains suboptimal. The Hippo family members play critical roles in tumorigenesis and inflammatory responses and have been reported to have critical prognostic significance in many cancer types. The prognostic roles and functions of Hippo-related genes and miRNA expression in OC have not yet been studied. In the current study, we comprehensively explored the expression patterns, prognostic values (PFS), genetic alterations, and potential functions of different Hippo members based on a variety of large online databases. Dysregulation of the Hippo pathway could be an important factor in the poor prognosis of ovarian cancer [34], a fact also confirmed by the present study.

**Figure 8:**
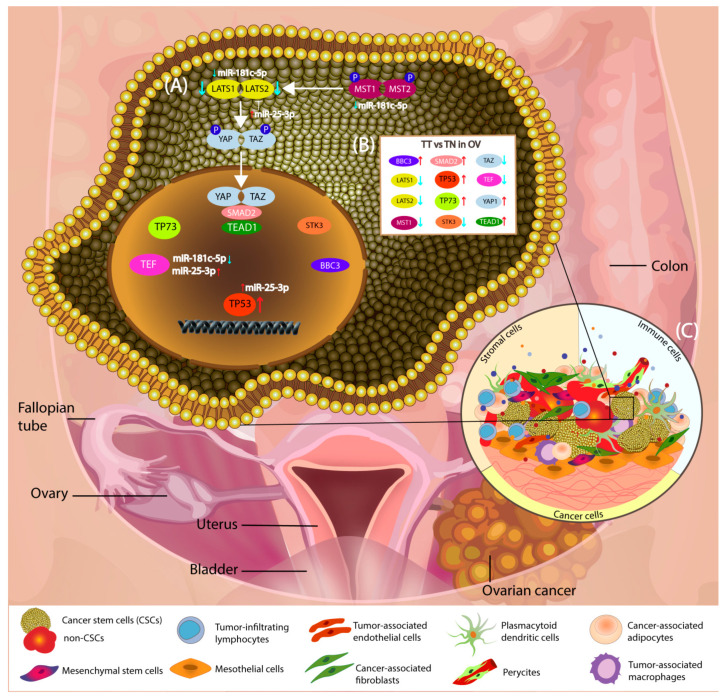
Schematic representation of Hippo signaling pathway in OC (**A**) The main genes involved in Hippo signaling as regards OC and the associated miRNAs. As shown, miR-25-3p target TP53, TEF, LATS1, LATS2, and miR-181c target TEF, LATS1, LATS2, and MST1, respectively. (**B**) Relative expression levels of the main genes involved in Hippo signaling pathway in OC, TT (tumor tissue) versus TN (normal tissue) (**C**) schematic representation of the OC tumor microenvironment.

The Hippo genes were shown to promote characteristics such as self-renewal, metastatic potential, and chemoresistance [35], displaying a poor prognosis in OC [26], a fact also sustained by the present study. In this study, we focused on the understanding of the molecular mechanisms of this family of genes and targeting miRNAs (Figure 8), paving the way for further studies.

MST1/2 activation results in the phosphorylation and activation of their direct substrates LATS1/2 [36]. LATS1 and LATS1 kinase modules are strongly conserved over evolution [37], and our study reveals the downregulation of these kinases. Understanding LATS-mediated tumor suppression will probably facilitate tools for early detection, prognosis, and treatment of OC [37]. LATS1 expression levels might be a valuable survival indicator in ovarian serous carcinoma [38]. Both LATS1 and LATS2 expression levels significantly correlated with recurrence and stage [38], confirmed for LATS2 also by the present data.

miRNA has an important role in the interplay between the Hippo pathway and other signaling pathways such as the MAPK, Notch, Wnt, and TGFβ pathways [19,37,39]. We should not underestimate the mutational effect on key Hippo genes that may affect the crosstalk with other signaling pathways [24]. In the modulation of the crosstalk, among them, the TP53 gene plays an important role [40]. The TP53 gene is frequently mutated in human cancers [41], including OC, where it was observed a direct connection between the TP53 mutational status and evolutionary conservation [5]. This may be related to the fact that the functional status of TP53 proteins dictates the subcellular localization, protein stability, and transcriptional activity of the core component of the Hippo pathway, YAP1. YAP1 and TP53 pathways are critical protectors of genome integrity in response to DNA damage. Interestingly, the YAP1 gene was not altered in OC [40].

The regulatory networks of TP53 and Hippo pathways are connected in a highly context-specific manner [39], affecting key cellular processes related to cell proliferation, apoptosis, or invasion [42,43,44]. OC patients presenting different mutated TP53 proteins show different chemotherapy responses and survival outcomes [42,45]. TP53 interacts directly with the LATS2 promoter to induce LATS2 expression [37].

The main TP53 target is represented by miR-34a, a transcript that has a powerful tumor suppressor role related to cell proliferation, particularly in advanced stages [43,46]. It was demonstrated that miR-34 family members are frequently downregulated in OC [43,44,46], in both TP53 wild-type and mutant subgroups [43,46], being inversely expressed with TP53 as shown in our study. miR-181 family members represent highly controversial transcripts, being overexpressed in OC and related to epithelial to mesenchymal transformation and resistance [47], including miR-181c [48]. Downregulation of miR-181c was also confirmed in our study. Additionally, overexpression of miR-25-3p was observed. An increased expression level for miR-25-3p is related to an unfavorable prognostic, indicative that this transcript holds prognostic value in OC [49]. Other studies revealed that miR-25-3p overexpression promoted OC cell proliferation and motility by targeting LATS2 [50] or apoptosis by targeting BIM [51]. This emphasizes the oncogenic potential of miR-25-3p in OC [50]. miR-25-3p overexpression promotes tumor metastasis by activation of EMT [52], a key mechanism involved in invasion and metastasis [53]. Hence, miR-25-3p can be considered as a cancer-specific transcript in OC, having prognostic value along with miR-7, miR-16, miR-93, miR-182, miR-376a, and miR-429 [52].

Hippo signaling provides multiple interactions with coding and non-coding genes that are regulated by a variety of mechanisms. Knowledge of the interconnections between the Hippo signaling pathway and other biological processes that result in tumorigenesis might promote the development of new therapeutic strategies in OC [54].

## 5. Conclusions

In summary, by using online databases, we accessed the expression levels and determined the prognostic roles of key Hippo signaling genes that were validated on an additional patient cohort.

These results indicate that key Hippo signaling genes and direct miRNAs (miR-25-3p and miR-181c-5p) have important prognostic value in OC. A better understanding of the heterogeneity and complexity of OC is needed to develop tools for new therapeutic strategies and accurately assess prognosis.

## Figures and Tables

**Table 1:** Clinical data of ovarian cancer (OC) patients.

Study ID	TT	TN	Age	Relapse	PFS (Months)	OS (Months)	FIGO Stage
3	Yes	N/A	75	0	6.9	33.5	IIIC
4	Yes	N/A	48	0	8.2	7.8	IVA
6	Yes	N/A	62	0	1	1.5	IIIC
7	Yes	N/A	59	1	16.6	16.6	IIIC
9	Yes	N/A	67	1	10.6	17	IIIC
13	Yes	N/A	53	1	10.5	21	IIIC
14	Yes	N/A	54	0	47.1	47.1	IIIC
15	Yes	N/A	48	1	25	39.6	IIIC
16	Yes	Yes	61	0	9.9	17.7	IIIC
20	Yes	Yes	69	0	8.4	8.4	IIB
22	Yes	Yes	67	1	9.7	10.8	IVA
23	Yes	Yes	46	0	13.4	13.4	IVA
24	Yes	Yes	49	1	14.2	23.4	IIIC
25	Yes	Yes	54	1	13.5	33	IIIC
30	Yes	Yes	34	0	24.3	24.3	IIIA
35	Yes	Yes	22	0	0.2	0.2	N/A
40	Yes	Yes	42	0	3.7	3.7	IA
43	Yes	Yes	43	1	6.6	38.6	IIIC
46	Yes	Yes	63	1	14.8	25.8	IIIA
52	Yes	Yes	75	0	8.5	8.5	IC
53	Yes	Yes	54	0	38.8	38,8	N/A
54	Yes	Yes	44	1	28	42.7	IVA
55	N/A	Yes	50	0	1.3	1.3	IVB
57	Yes	Yes	73	0	2.6	2.7	IVB
59	Yes	N/A	41	0	24,8	24.8	IA
60	N/A	Yes	62	0	3,2	3.2	IB
61	Yes	N/A	68	0	25	25	IC
62	Yes	Yes	63	0	21.9	21.9	IIIB
65	Yes	N/A	63	0	0.3	0.3	IIIA
69	Yes	Yes	57	1	12,4	27.2	IIIC
71	Yes	Yes	50	0	15	15	IIIA

TT = tumor tissue, TN = normal tissue, N/A = not available.

**Table 2:** Correlation of key Hippo genes with PFS according to tumor grade, hazard ratio, and 95% confidence interval.

Gene	Affymetrix IDs	Tumor Grade	No. of Patients		HR	95% CI	*p*-Value
			Low Expression	High Expression			
LATS1	227772_at	I + II	64	51	1.51	0.93–3.11	0.26
		III + IV	151	363	0.85	0.69–1.05	0.13
LATS2	227013_at	I + II	50	65	0.57	0.28–1.19	0.13
		III + IV	300	164	1.57	1.29–1.92	8.1 × 10^−6^
MST1	213382_at	I + II	55	108	0.56	0.32–1	0.046
		III + IV	702	379	1.07	0.93–1.24	0.35
TP53	201746_at	I + II	40	123	0.48	0.27–0.87	0.014
		III + IV	582	499	0.82	0.71–0.94	0.0057
TAZ	202133_at	I + II	63	100	0.54	0.3–0.95	0.031
		III	569	512	1.15	0.99–0.32	0.06
TEF	225840_at	I + II	72	43	0.5	0.23–1.09	0.075
		III + IV	175	319	1.39	1.13–1.72	0.0022

**Table 3:** Correlation of key Hippo genes with PFS according to TP53 status, hazard ratio, and 95% confidence interval.

Gene	Affymetrix IDs	TP53 Mutation Status	No. of Patients		HR	95% CI	*p*-Value
			Low Expression	High Expression			
LATS1	227772_at	Wild-type	5	14	1.78	0.56–5.63	0.32
		Mutant	76	48	1.39	0.95–2.03	0.093
LATS2	227013_at	Wild-type	7	12	5.13	1.35–19.5	0.0091
		Mutant	55	69	1.68	1.15–2.45	0.0067
MST1	223382_at	Wild-type	26	58	0.62	0.35–1.13	0.11
		Mutant	263	220	1.23	0.98–1.54	0.069
TP53	201746_at	Wild-type	43	41	0.57	0.33–0.99	0.043
		Mutant	317	166	1.34	1.06–168	0.012
TAZ	202133_at	Wild-type	214	269	1.22	0.97–1.53	0.089
		Mutant	39	45	1.34	0.79–2.28	0.28
TEF	225840_at	Wild-type	4	15	4.17	0.92–18.88	0.047
		Mutant	66	58	1.59	1.09–2.31	0.015

## Supplementary Materials

The following are available online at https://www.mdpi.com/2075-4418/11/2/344/s1, Figure S1: UALCAN analyses of key Hippo genes in OC.
