# Supplementary material for: Comprehensive Analysis of the Expression of Key Genes Related to Hippo Signaling and Their Prognosis Impact in Ovarian Cancer
Source: Diagnostics (Basel). 2021 Feb 19;11(2):344. doi: 10.3390/diagnostics11020344 (PMC7922135; doi:10.3390/diagnostics11020344)
Supplement: Supplementary file 1 [file diagnostics-11-00344-s001.pdf]

Supplementary Material

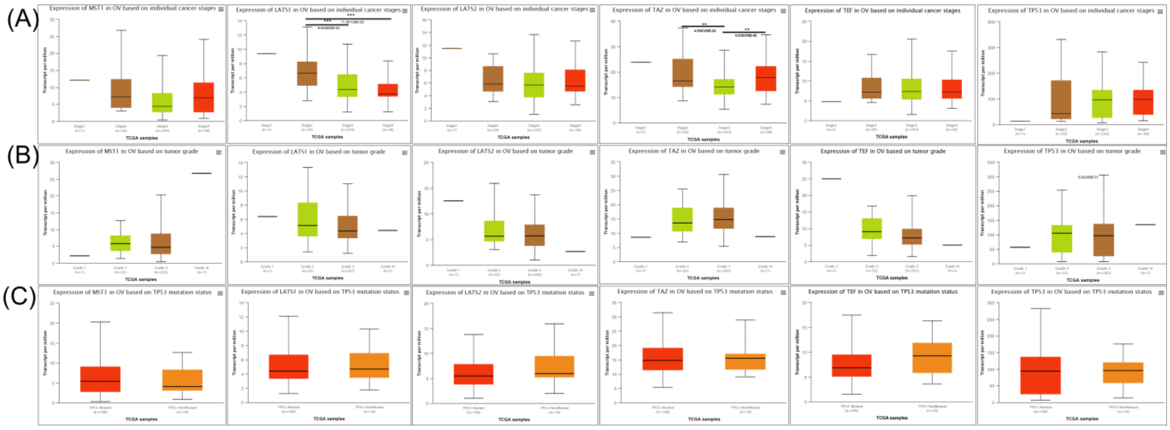

Figure S1. UALCAN analyses of key Hippo genes in OC. Expression levels according to (A) stage (stage I, n=1 black color; stage II, n=20, brown color; stage III n=243, green color; stage IV, n=38, red color); (B) tumor grade (grade I, n=1, black color; grade II, n=32 green color; grade III n=262, brown color; grade IV, n=1, black color) and (C) TP53 mutational status (TP53 mutant, n=199, red color; TP53 non-mutant, n=19, orange color).
